# Neoadjuvant radiotherapy for resectable retroperitoneal sarcoma: a meta-analysis

**DOI:** 10.1186/s13014-022-02159-3

**Published:** 2022-12-28

**Authors:** Xiangji Li, Ruihan Dong, Mengmeng Xiao, Li Min, Chenghua Luo

**Affiliations:** 1 Department of Retroperitoneal Tumor Surgery, Peking University International Hospital, 1 Shengmingyuan Road, Changping District, Beijing, People’s Republic of China; 2 Department of Gastroenterology, National Clinical Research Center for Digestive Disease, Beijing Digestive Disease Center, Beijing Key Laboratory for Precancerous Lesion of Digestive Disease, Beijing Friendship Hospital, Capital Medical University, Beijing, People’s Republic of China; 3 Beijing Shijitan Hospital, Capital Medical University, Beijing, People’s Republic of China

**Keywords:** Retroperitoneal sarcoma, Neoadjuvant radiotherapy, Meta-analysis, Soft tissue sarcoma

## Abstract

**Background:**

Neoadjuvant radiotherapy (NRT) for resectable retroperitoneal sarcoma (RPS) has been shown to be systematically feasible. Whether NRT has equivalent or better clinical effects compared to surgery alone for RPS patients remains controversial.

**Methods:**

We performed a systematic literature search of PubMed, Web of Science, Embase, ASCO Abstracts, and Cochrane library databases for studies in humans with defined search terms. Articles were independently assessed by 2 reviewers, and only randomized controlled trials and cohort studies were included. The hazard ratios (HRs) of overall survival (OS), recurrence-free survival (RFS), and local recurrence (LR) were extracted from included studies. Heterogeneity among study-specific HRs was assessed by the Q statistic and I^2^ statistic. Overall HR was assessed by random-effects or fixed-effects models. Publication bias was tested by Begg’s tests, and the quality of each study was assessed with the Newcastle Ottawa Scale.

**Results:**

A total of 12 eligible studies with 7778 resectable RPS patients were finally included in this study. The pooled analysis revealed the distinct advantages of NRT as compared to surgery alone, including longer OS (HR = 0.81, *P* < 0.001), longer RFS (HR = 0.58, *P* = 0.04), and lower LR (HR = 0.70, *P* = 0.03). No evidence of publication bias was observed.

**Conclusion:**

NRT is likely to be beneficial for resectable RPS patients in terms of OS and RFS*.* However, more multicenter clinical trials are needed to confirm these findings.

**Supplementary Information:**

The online version contains supplementary material available at 10.1186/s13014-022-02159-3.

## Introduction

Retroperitoneal sarcomas (RPS) are rare malignant tumors that occur in the retroperitoneum, accounting for 20% of all soft tissue sarcomas (STS) in adults [1]. They tend to fill the abdominal cavity and encapsulate or invade the surrounding organs when initially diagnosed due to insidious onset and atypical symptoms. Surgery remains the only potentially effective curative approach, and concomitant multi-visceral resection often is performed to achieve better local control and to prolong the overall survival (OS) [2–4]. However, the special anatomical structure of the retroperitoneum, heterogeneity of RPS with different biological behavior, and oncological risks according to subtypes render a homogeneous surgical treatment difficult. Currently, neoadjuvant radiotherapy (NRT) has been applied to the treatment of RPS and presents a number of conceptual advantages over traditional surgical treatment. Preoperative radiotherapy is able to define the treatment field with high accuracy, minimizing the toxicity of adjacent structures caused by tumor mass displacement [5, 6], while maximizing R0 resection rates and minimizing the risk of local recurrence (LR) or peritoneal seeding [7, 8]. However, data supporting neoadjuvant radiotherapy in RPS are limited, and justification for its use has been extrapolated from its established role in extremity STS [9, 10]. To date, only one completed trial randomly (EORTC-62092: STRASS) assigned 266 patients, comparing 3D conformal or intensity-modulated radiotherapy (50.4 Gy, in 28 daily fractions of 1.8 Gy) plus surgery with surgery alone [11]. In this trial, patients who received NRT did not have improved recurrence-free survival (RFS) but more frequent grade 3–4 adverse events than control patients. The results of other retrospective studies, including analyses of large national databases, that investigate the role of NRT are not consistent [12–22]. In addition. Chinese consensus guidelines for diagnosis and treatment of primary retroperitoneal soft tissue sarcoma (2019 edition) suggest that local radiotherapy is not recommended for every patient with resectable RPS (level B evidence, level 2 recommendation) [23]. In the absence of a high level of evidence, whether to incorporate NRT into the clinical treatment of RPS has been controversial. To address a gap in knowledge, we aimed to evaluate the impact of NRT on RPS via this meta-analysis.

## Methods

### Database and bibliography retrieval

PubMed, Web of Science, Embase, ASCO Abstracts, and Cochrane library databases were searched for eligible studies published between January 2000 and January 2020 following the PRISMA statement (preferred reporting items for systematic reviews and meta-analysis). The search terms were: Retroperitoneal neoplasms OR Retroperitoneal sarcomas OR Retroperitoneal soft tissue sarcomas; Neoadjuvant therapy OR Neoadjuvant radiotherapy OR Preoperative radiotherapy; Surgery OR Radiosurgery. In addition, we also identified eligible studies from previous related reviews.

### Inclusion and exclusion criteria

Studies were included based on the following criteria: (1) it was a randomized clinical trial (RCT) or comparative study of NRT versus surgery for resectable RPS patients; (2) RPS confirmed by pathological biopsy; (3) at least one of the following information was reported: the hazard ratio (HR) and 95% confidence interval (CI) of LR, RFS, and OS, Kaplan–Meier curve and other valid data to calculate HR. The exclusion criteria included: (1) reviews, letters, editorials, or non-comparative studies; (2) HR and its 95% CI unable to be calculated based on available data; (3) the cases or the groups in the study were fewer than 20 and five respectively; (4) repeated reports by the same institution; (5) Newcastle Ottawa Scale (NOS) less than 6 [24]; (6) non-human studies. Finally, this meta-analysis was conducted by reported outcomes indications from included studies and not individual data.

### Data extraction

Two authors (Li and Dong) independently extracted data from eligible literature. Disagreements between authors were resolved by consensus and invited senior scholars to interpret if the differences were still controversial after discussion. The information extracted included: first author, year of publication, patient source, intervention, number of patients, type of study, and outcome. Study quality was evaluated by NOS which includes nine criteria to assess both randomized and non-randomized comparative studies. A study was considered of high quality if it scored 7 points or higher.

### Statistical analysis

Meta-analysis was performed by Review Manager version 5.4 (Cochrane Collaboration, London, UK). The heterogeneity was determined by χ^2^ based on Q statistic and *I*^*2*^ statistic. When heterogeneity is significant (*P* < 0.05, *I*^*2*^ > 50%), a random effect model was used to merge the pool HR and a sensitivity analysis was performed subsequently. Otherwise, a fixed effect model was used. Publication bias was assessed by StataCorp version 15.1 (College Station, TX 77845, USA) with Begg’s test.

## Results

### Search results and characteristics of eligible studies

**Fig. 1:**
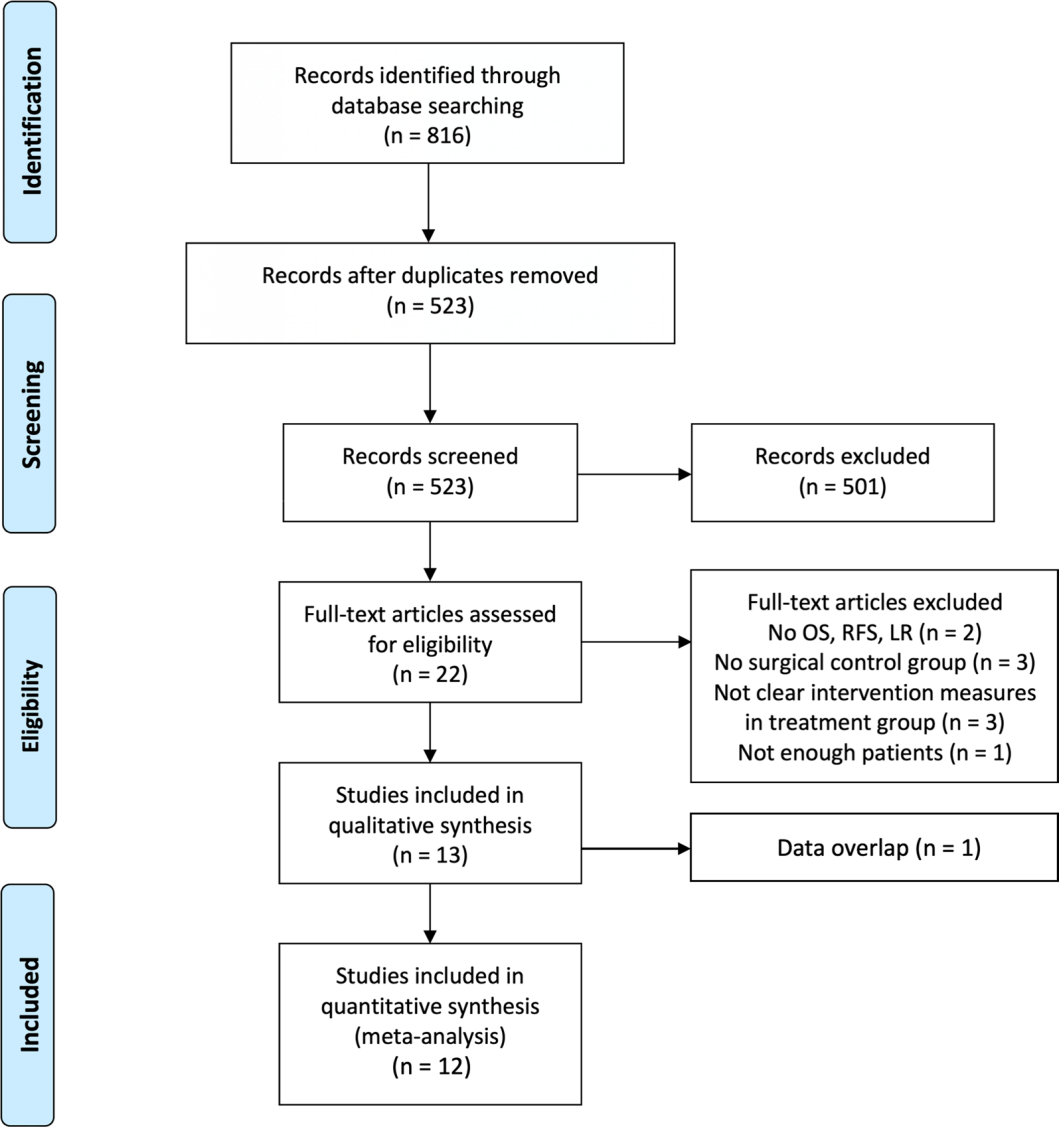
Preferred Reporting Items for Systematic Reviews and Meta-Analyses (PRISMA). Flowchart of studies included in the review with reasons for exclusion

A total of 816 relative references were identified from those databases, of which 285 were from PubMed, 386 were from Web of Science, 133 were from Embase, 8 were from Cochrane library, and the remaining four were from ASCO Abstracts. After selection according to the inclusion/exclusion criteria, 12 studies including one RCT and 11 retrospective cohort studies (RCSs) were eligible for meta-analysis (Fig. 1). Among them, five studies divided participants into the NRT group and surgery group with propensity score-matched (PSM). OS, RFS, and LR were used as outcome indicators in all included studies, and the quality scores with NOS were between seven and eight (see Additional file 1: Table S1). The characteristics of those included studies were shown in Table 1.

**Table 1:** Characteristics of included studies

Study (Author, Year)	Patient source	Type of study	Study period	Male		Median follow up (Months)	Number of patients (RT + Sur/Sur)	Age (years)		Histological subtypes	
				RT + Sur (%)	Sur (%)			RT + Sur	Sur	RT + Sur (%)	Sur (%)
Chouliaras et al. 2019 [15]^a^	USA	RCS	2000–2016	37.0	37.0	31.4^c^	46/46	59.3 (50.5–68.5)^d^	64.6 (51.9–78.9)^d^	LPS (26.1) LMS (34.8) UPS (21.7) Other (17.4)	LPS (30.4) LMS (26.1) UPS (28.2) Other (15.3)
Turner et al. 2019 [12]	Canada	RCS	1990–2014	42.5	38.7	90.0^c^	40/62	56.7^b^	60.0^b^	LPS (32.5) Other (72.6)	LPS (27.4) Other (67.5)
Ecker et al 2016 [17]^a^	USA	RCS	2004–2013	54.6	55.5	52.0 (30.9–75.1)^d^	174/173	$\le$ 63 (57.7%) 64.0–72 (25.3%) $\ge$ 72 (17.2%)	$\le$ 63 (52.0%) 64–72 (28.3%) $\ge$ 72 (19.7%)	NR	NR
Kelly et al 2015 [16]	USA	RCS	2003–2011	47	51	Treat 36.9^c^ Control 38.8^c^	32/172	57.0 (41.0–85.0)^c^	62.0 (26.0–92.0)^c^	LPS (59.4) LMS (25.0) MPNST (3.1) Other (12.5)	LPS (68.0) LMS (28.5) MPNST (1.7) Other (1.8)
Nussbaum et al 2016 [18]^a^	USA	RCS	2003–2011	56.0	54.0	Treat 42.0 (27.0–70.0)^d^ Control 43.0 (25.0–64.0)^d^	563/1126	59.2 (13.8)^b^	59.5 (14,5)^b^	LPS (37.4) LMS (27.2) MFH (8.0) MPNST (1.7) Other (25.7)	LPS (40.9) LMS (27.6) MFH (6.7) MPNST (0.7) Other (24.1)
Lane et al. 2015 [22]	USA	RCS	1990–2011	37.5	41.7	Treat 84.4 (58.6–94.3)^d^ Control 25.0 (4.2–52.5)^d^	8/12	52.5 (47.0–62.2)^d^	48.5 (43.2–60.5)^d^	LPS (87.5) LMS (12.5) UPS (0.0) Other (0.0)	LPS (41.6) LMS (41.7) UPS (0.0) Other (16.7)
Bonvalot et al. 2020 [11]^a^	Europe USA Canada	RCT	2012–2017	53.0	50.0	43.1 (28.8–59.2)^d^	133/133	61.0 (52.0–68.0)^d^	61.0 (53.0–67.0)^d^	LPS (73.6) LMS (12.0) Other (14.4)	LPS (75.2) LMS (16.5) Other (8.3)
Ma et al. 2020 [19]^a^	USA	RCS	2006–2015	55.3	55.2	48.7 (27.6–76.8)^d^	844/844	$<$ 65 (55.7%) $\ge$ 65 (44.3%)	$<$ 65 (56.5%) $\ge$ 65 (43.5%)	LPS (47.1) LMS (27.3) MFH (2.7) MPNST (1.5) Other (21.4)	LPS (45.6) LMS (26.5) MFH (2.1) MPNST (1.9) Other (23.9)
Bremjit et al. 2014 [14]	USA	RCS	2000–2013	41.7		31.8 (1.4–257.3)^c^	40/92	54.8 (25.8–88.5)^c^		LPS (60.6) LMS (22.0) Other (17.4)	
Snow et al. 2018 [20]	Australia	RCS	2008–2016	61.0		NR	62/32	59.0 (18.0–86.0)^c^		LPS (53.2) LMS (18.1) UPS (7.4) Other (21.3)	
Bonvalot et al. 2009 [13]	France	RCS	1985–2005	51.8		52.8 (12.0–216.0)^c^	122/260	57.0 (14.0–87.0)^c^		LPS (49.7) LMS (17.8) MFH (8,9) Other (23.6)	
Berger et al. 2018 [21]	USA	RCS	2004–2013	47.6		NR	272/2490	62.9 (11.2)^b^		NR	

Study (Author, Year)	Tumour size (cm)		Surgical margins		Radiotherapy Dose (Gy)	Other radiotherapy	Chemotherapy (Y/N)	Outcome HR (95%CI)		NOS score
	RT + Sur	Sur	Treat (Neg/Tol)	Control (Neg/Tol)						
Chouliaras et al. 2019 [15]^a^	13.75 (9.5–19.0)^d^	15.25 (10.2–21.0)^d^	26/46	23/46	NR	NR	Y (Treat: 32.6%; Control: 10.9%)	OS	1.14 (0.60–2.17)	8
								RFS	0.98 (0.52–1.84)	
								LR	1.18 (0.51–2.74)	
Turner et al. 2019 [12]	136.5^c^	184.0^c^	29/40	19/62	49.0^c^	NR	N	OS	0.42 (0.19–0.90)	8
								RFS	0.43 (0.24–0.79)	
Ecker et al. 2016 [17]^a^	$\le$ 10 (13.2%) 10–20 (42.0%) $\ge$ 20 (44.8%)	$\le$ 10 (10.4%) 10–20 (45.7%) $\ge$ 20 (43.9%)	NR	NR	50.0 (45.0–50.4)^c^	NR	Y (Treat: 13.2%; Control: 11.0%)	OS	0.64 (0.42–0.99)	8
Kelly et al. 2015 [16]	< 18 (63.0%) $\ge$ 18 (38.0%)	< 18 (48.0%) $\ge$ 18 (52.0%)	14/32	105/172	50.4 (14.0–62.0)^c^	IOERT (Treat: 47.0%)	N	DSS	0.52 (0.12–2.22)	8
Nussbaum et al. 2016 [18]^a^	15.4 (11.9)^b^	16.0 (11.9)^b^	NR	NR	NR	NR	N	OS	0.70 (0.58–0.84)	8
Lane et al. 2015 [22]	$<$ 5 (0%) 5–10 (62.5%) 10–15 (25.0%) $>$ 15 (12.5%)	$<$ 5 (27.3%) 5–10 (9.1%) 10–15 (9.1%) $>$ 15 (54.5%)	3/8	4/12	45.0 (32.4–56.2)^c^	IOETR (Treat: 75.0%)	Y (Treat: 12.5%; Control: 41.7%)	OS	0.30 (0.11–0.82)	8
								RFS	0.34 (0.17–0.69)	
Bonvalot et al. 2020 [11]^a^	16.0 (11.1–21.0)^d^	16.7 (12.4–21.0)^d^	114/119	122/128	50.4	NR	N	RFS	1.01 (0.71–1.44)	8
Ma et al. 2020 [19]^a^	NR	NR	568/844	552/844	NR	NR	N	OS	0.88 (0.77–0.99)	8
Bremjit et al. 2014 [14]	18.5 (3.0–55.0)^c^		Neg/Tol: 60/66		NR	NR	Y (21.2%)	OS	0.70 (0.30–1.60)	7
Snow et al. 2018 [20]	130.0 (20.0–420.0)^c^ 130.0 (76.7–190.0)^d^		Neg/Tol: 83/94		NR	NR	N	OS	1.00 (0.40–2.70)	7
								RFS	0.33 (0.13–0.84)	
Bonvalot et al. 2009 [13]	18.0 (3.0–60.0)^c^		Neg/Tol:176/382		45.0(10.0–66.0)^c^	IOETR (4.7%) Dose (10–18)	Y (38.0%)	LR	0.64 (0.45–0.90)	7
Berger et al. 2018 [21]	19.9 (11.9)^b^		Neg/Tol:1445/2762		NR	post-RT (19.9%)	N	OS	0.89 (0.69–1.14)	7

*CI* confidence interval, *DSS* disease-specific survival, *HR* hazard ratio, *IOERT* intraoperative radiation therapy, *MFH* malignant fibrous histiocytoma, *MPNSTL* malignant peripheral nerve sheath tumor, *LMS* leiomyosarcoma, *LPS* liposarcoma, *LR* local recurrence, *Neg* negative, *N* no, *NR* not reported, *OS* overall survival, *Pos* positive, *RCS* retrospective cohort study, *RCT* randomized clinical trial, *RFS* recurrence-free survival, *RT* radiotherapy, *Sur* surgery, *Tol* total, *UPS* undifferentiated pleomorphic sarcoma, *Y* yes

LR is defined as a new tumor lesion detected near the primary lesion after surgical resection

^a^Propensity score matched (PSM)

^b^Data are mean or mean (SD)

^c^Data are median or median (range)

^d^Data are median (IQR)

### Meta-analysis of OS

**Fig. 2:**
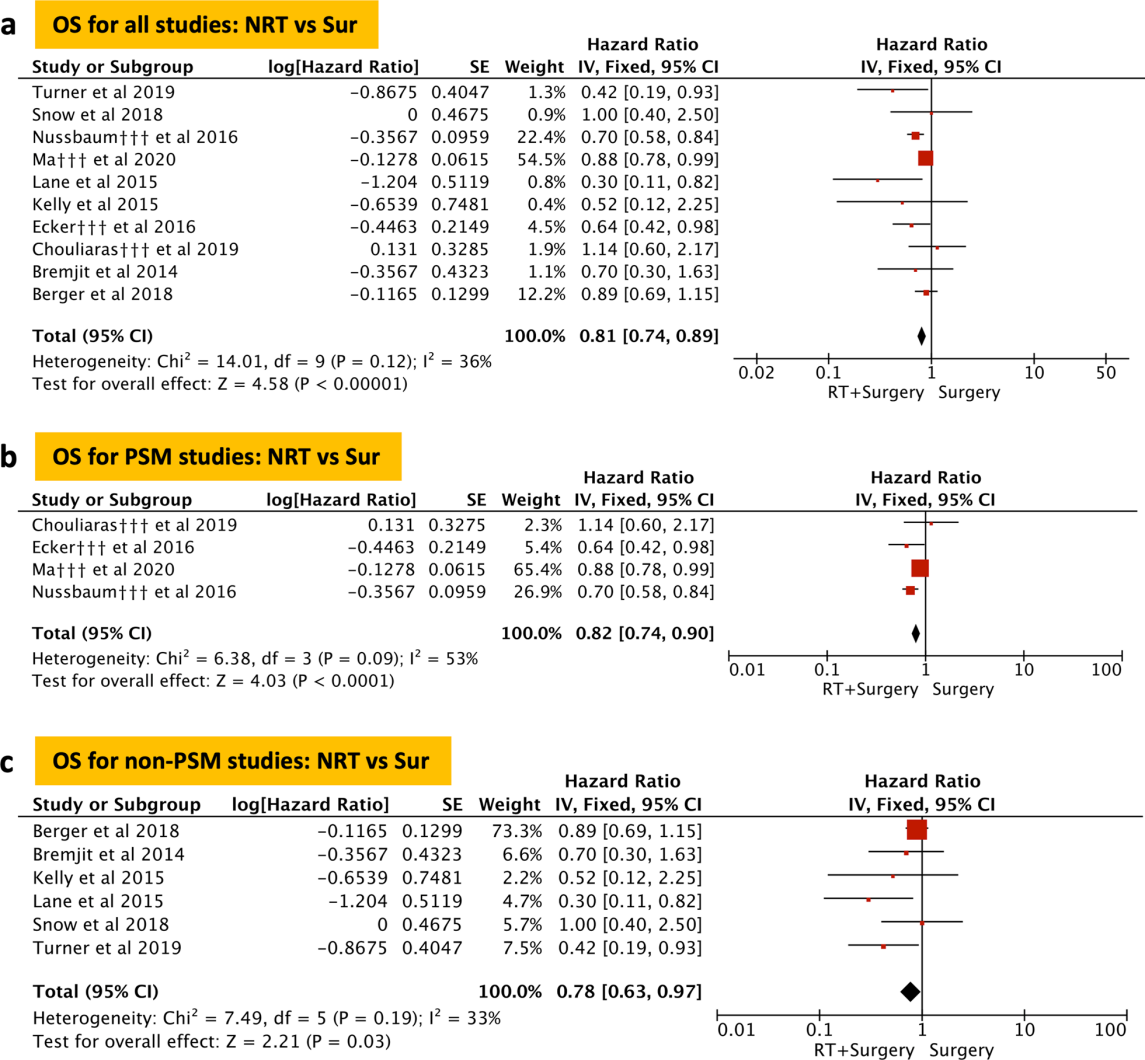
Overall survival (OS). Forest plot and pooled analysis of hazard ratio for OS of all studies (**a**), studies with propensity score matched (PSM) (**b**), and studies without PSM (**c**). The area of symbols reflects the weight of studies, NRT = neoadjuvant radiotherapy, Sur = surgery, ††† = propensity score matched

OS was reported in 10 of the 12 included studies and 7130 patients were included. 2081 in the NRT group and 5049 in the surgery group. The fixed-effect model was used (Fig. 2a), and the pooled HR showed that the NRT could significantly improve the OS of RPS compared to the surgery alone (HR = 0.81, *P* < 0.001). Subsequently, we conducted separate analyses of the studies with and without PSM, and the results revealed that there were no significant differences in the pooled analysis of studies with PSM (NRT vs surgery, HR = 0.82, *P* < 0.001; Fig. 2b) and without PSM (NRT vs surgery, HR = 0.78, *P* = 0.03; Fig. 2c).

### Meta-analysis of RFS

**Fig. 3:**
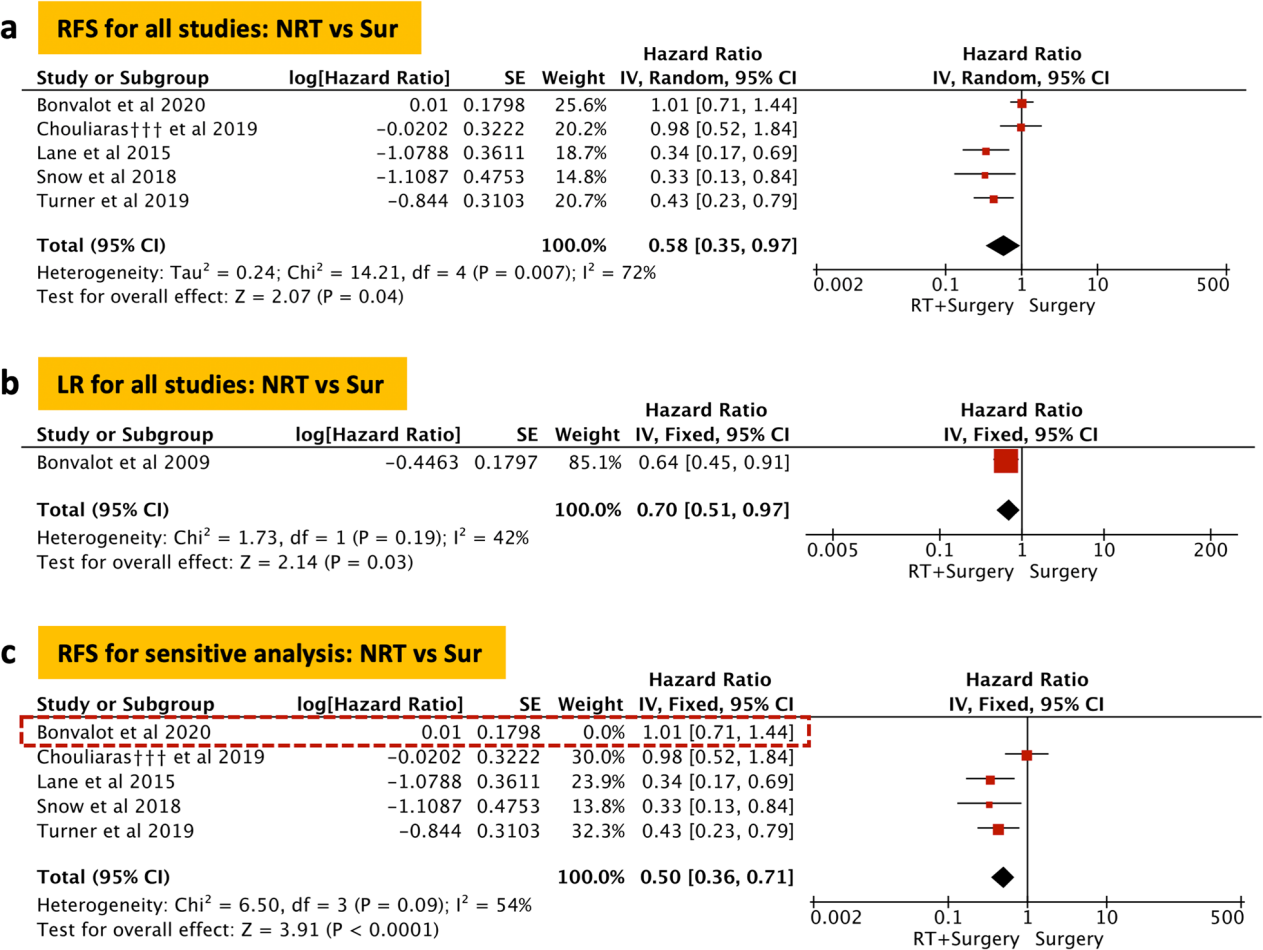
Recurrence-free survival (RFS) and local recurrence (LR). Forest plot and pooled analysis of hazard ratio for RFS (**a**) and LR (**b**). Forest plot and sensitivity analysis of hazard ratio for RFS (**c**). The area of symbols reflects the weight of studies, NRT = neoadjuvant radiotherapy, Sur = surgery, ††† = propensity score matched

The meta-analysis included 5 studies and 574 patients. There was significant statistical heterogeneity in these included studies. The random-effect model was used and a notable statistical difference was found in the RFS between the two groups (NRT vs surgery, HR = 0.58, *P* = 0.04; Fig. 3a).

### Meta-analysis of LR

Two eligible studies were included, including 168 patients in the NRT group and 306 patients in the surgery group. No significant statistical heterogeneity was found in the two studies (Fig. 3b). The result showed NRT group had lower LR than the surgery group (NRT vs surgery, HR = 0.70, *P* = 0.03).

### Sensitivity analysis and publication bias

As mentioned above, there was notable heterogeneity in the analysis of RFS, thus we conducted a sensitivity analysis by excluding the study of high heterogeneity (Table 2). We found Bonvalot [11] et al. (2020) was the main source of heterogeneity. After excluding the heterogeneous factor, we used a fixed-effect model to conduct pooled analysis, and the result indicated RFS was also obviously improved in the NRT group (NRT vs surgery, HR = 0.50, *P* < 0.001; Fig. 3c).

**Table 2:** Summary of results

Categories	Studies	Patients		Model	HR (95%CI)			Heterogeneity		
		NRT	Sur		value	z	*P* value	Chi^2^	I^2^	*P* value
**OS**										
Neoadjuvant radiotherapy	10	2081	5049	Fixed	0.81 (0.74–0.89)	4.58	< 0.001	14.01	36%	0.12
Neoadjuvant radiotherapy_non-PSM	6	454	2860	Fixed	0.78 (0.63–0.97)	2.21	0.03	7.49	33%	0.19
Neoadjuvant radiotherapy_PSM	4	1627	2189	Fixed	0.82 (0.74–0.90)	4.03	< 0.001	6.38	53%	0.09
**RFS**										
Neoadjuvant radiotherapy	5	289	285	Random	0.58 (0.35–0.97)	2.07	0.04	14.21	72%	0.007
Sensitivity analysis	4	156	152	Fixed	0.50 (0.36–0.71)	3.91	< 0.001	6.50	54%	0.09
**LR**										
Neoadjuvant radiotherapy	2	168	306	Fixed	0.70 (0.51–0.97)	2.14	0.03	1.73	42%	0.19

*LR* local recurrence,* NRT* neoadjuvant radiotherapy,* OS* overall survival,* PSM* propensity score matched,* RFS* recurrence-free survival,* Sur* surgery

Publication bias was evaluated by Begg’s test, and no significant statistical bias was found in the included studies (see Additional file 1: Fig. S1 and Table S2).

## Discussion

High local recurrence rates (> 50%) and low overall survival rates (40%-60%) have been major challenges for clinicians in managing patients with RPS [25–27]. Radiotherapy is a potent approach to reduce postoperative recurrence, which has been proven in a variety of malignant tumors. Diamantis et al. [28] reported a systematic review and meta-analysis comparing perioperative radiotherapy with surgery alone demonstrating that both the OS and RFS could be elongated by radiotherapy (OR = 0.69, *P* = 0.005; OR = 0.19, *P* < 0.001).

According to the current experience of RPS management, postoperative radiotherapy could cause unwanted injury of sensitive abdominal organs, since those organs, especially the small bowel, easily falls into the space occupied by removed sarcoma mass and are exposed to high doses of irradiation [8]. Thus, preoperative radiotherapy was proposed as an alternative, but there is no clear evidence on whether preoperative radiotherapy could improve the prognostic outcomes of RPS patients.

Previously, we mentioned a randomized study comparing the curative effect of NRT versus surgery alone in patients with RPS [11]. It is the first large, international, randomized trial in primary, localized RPS that has been successfully completed. Although this trial is negative, with similar abdominal RFS (HR = 1.01; *P* = 0.95) and OS (HR = 1.16; *P* = 0.65) in both groups at 3 years of follow-up, it shows that key questions in rare cancer can be addressed through multi-institutional collaboration. The randomization offsets selection biases inherent in retrospective series such as the smaller tumors, in more favorable locations, easier to resect, and resected in academic centers. Therefore, this conclusion replaces the heterogeneous approach to NRT for RPS, whereby its use varied considerably based on investigator and institutional biases. In addition, adverse events were assessed in this trial, with more grade 3–4 adverse events in the NRT than surgery alone group (98/127 vs 1/128 for lymphopenia, 15/127 vs 10/128 for anemia, and 15/127 vs 5/128 for hypoalbuminemia), and more serious adverse events in NRT than surgery alone group (30/127 vs 13/128). However, a more prominent limitation in this trial is the controversial definition of abdominal recurrence, which leads to instability of the analysis results. In the post-hoc, exploratory of patients with liposarcoma histology, there was no significant difference in RFS between NRT and surgery alone [well-differentiated liposarcoma (HR = 0.69, 95%CI 0.33–1.46); dedifferentiated liposarcoma (HR = 0.92, 95%CI 0.53–1.61)]. However, in the first and second sensitivity analyses, NRT potential improved RFS compared to surgery alone (HR = 0.64, 95%CI 0.40–1.01; HR = 0.62, 95%CI 0.38–1.02). Therefore, more clinical trials should be performed to evaluate the efficacy of NRT in the treatment of resectable retroperitoneal liposarcoma.

In our meta-analysis, our pooled analysis revealed the distinct advantages of NRT versus surgery alone, including a longer OS, a longer RFS, and a lower LR. However, some limitations to be aware of when the results are considered. First, interventions in the treatment group in some studies were not limited to preoperative radiotherapy, several studies also have involved intraoperative radiation therapy and chemotherapy (Details are shown in Table 1). In order to minimize these confounding factors, we extracted the HR of NRT from the multivariate COX regression analysis (Details are shown in Additional file 1: Table S3). Second, strictly control of patient selection bias was difficult to achieve in the comparison of OS and RFS in all included studies [one RCT (8.3%) and eleven RCSs (91.7%)] due to inconsistencies in radiation dose, surgical margin, tumor grade, and pathological subtype of patients. Therefore, these inconsistent variables should be fully considered in optimal study design, the unification of radiotherapy dose and surgical margins is the prerequisite for contrasting the efficacy of NRT versus surgery for RPS patients, and directly determines the reliability of the pooled results. Investigators should at least acquire sufficient data for further analysis of pathological subtypes. Particularly for liposarcomas (well-differentiated liposarcoma and dedifferentiated liposarcoma) and leiomyosarcomas, as they are major components of RPS and exhibit significant biological heterogeneity. Although the previous RCT reported that NRT could not effectively improve the RFS of LMS compared with surgery alone (HR = 1.35, 95%CI: 0.55–3.32), the sample size in the study is too small to be convincing. (NRT vs Sur = 16 vs 22). Besides, it is worth noting that five studies (41.7%), including one RCT, used PSM, reducing data selection bias and decreasing the effect of confounding factors to some extent [11, 15, 17–19], and the pooled results of these PSM studies were consistent with those from non-PSM studies. Finally, there were only two studies in the subgroup analysis of LR. Although no major heterogeneity was found in this pooled analysis, it is still not sufficient to explain that NRT improves LR in RPS patients. Further subgroup analyses of RFS and OS by RPS subtype were also not performed due to a lack of data. It is noteworthy that several completed and ongoing studies could help refine which liposarcoma subtypes might benefit from radiotherapy, and we summarized the details in Table 3.

**Table 3:** Recent and ongoing trials of NRT in RPLS

Trial Name	Status	Primary Aims/Findings	Ref
Retroperitoneal SArcoma Registry: an International Prospective Initiative (RESAR) (NCT03838718)	Recruiting (12/2030)^a^	To assess the survival after surgery for primary RPS resection (Sarcoma subtype: primary RPS amenable to surgical resection)	NA
Proton or Photon RT for Retroperitoneal Sarcoma (NCT01659203)	Recruiting (08/2025)^a^	To determine the maximum tolerated dose (MTD) of preoperative IG-IMRT with simultaneously integrated boost to the high-risk margin of retroperitoneal sarcoma To determine the local control rate after the protocol treatment (IG-IMPT or IMRT MTD with simultaneously integrated boost to the high-risk margin) followed by surgical resection (Sarcoma subtype: primary RPS)	NA
Neoadjuvant Irradiation of Retroperitoneal Soft Tissue Sarcoma With Ions Retro-Ion (Retro-Ion) (NCT04219202)	Recruiting (05/2024)^a^	To evaluate the safety and feasibility of a hypofractionated, accelerated radiation approach based on the incidence of grade 3–5 NCI common terminology criteria for adverse events toxicity and/or termination of the planned therapy for any reason with neoadjuvant radiation with active beam guidance of the retroperitoneal sarcomas using protons or carbon ions before subsequent tumor resection (Sarcoma subtype: RPS which is resectable or marginally resectable)	NA
Surgery With or Without Radiation Therapy in Treating Patients With Primary Soft Tissue Sarcoma of the Retroperitoneum or Pelvis (NCT00091351)	Completed (02/2006)^b^	Compare progression-free survival of patients with primary soft tissue sarcoma of the retroperitoneum or pelvis treated with surgery with vs without preoperative radiotherapy (Sarcoma subtype: primary RPS)	NR
Preoperative Ultra-hypofractionated Radiotherapy Followed by Surgery for Retroperitoneal Sarcoma (NCT05224934)	Recruiting (12/2024)^a^	To investigate the feasibility and peri-operative complications of preoperative hypo-fractionated radiotherapy followed by surgery for retroperitoneal sarcoma (Sarcoma subtype: primary RPS)	NA

*IG-IMRT* image-guided intensity-modulated radiotherapy, *IMRT* intensity-modulated radiotherapy, *NA* not appliable, *NR* not reported, *RPS* retroperitoneal sarcoma

^a^Estimated study completion date

^b^Actual study completion date

Some scholars do not support the use of NRT for RPS due to concerns regarding delayed surgery and RT-associated toxicity. A recent study showed that no difference was found in LR and OS associated with the timing of surgical resection after EBRT [29], which indirectly supported the conduct of the RCT to compare NRT with surgery alone in RPS. We believed that more RCTs should be conducted in the future to provide more clear evidence for the efficiency of NRT on RPS management.

## Conclusion

Based on the above results, we believe that NRT is likely to be beneficial for resectable RPS patients in terms of OS and RFS*.* However, more multicenter clinical trials are needed to confirm these findings.

## Supplementary Information

**Additional file 1: Table S1.** The quality of enrolled studies. **Table S2.** Begg’s test for publication bias. **Table S3.** Univariate/Multivariate Cox proportional HR for outcomes in patients (NRT vs surgery). **Fig. S1.** Begg’s test for overall survival (OS) of all included studies (a), studies with propensity score matched (PSM) (b), and studies without PSM (c). Begg’s test for recurrence-free survival (RFS) in all included studies (d).

## Data Availability

The raw data supporting the conclusions of this article will be made available by the authors, without undue reservation.

## Abbreviations

CI: Confidence interval
HR: Hazard ratio
LR: Local recurrence
NRT: Neoadjuvant radiotherapy
NOS: Newcastle Ottawa scale
OS: Overall survival
PRISMA: Preferred reporting items for systematic reviews and mate-analysis
PSM: Propensity score-matched
RCS: Retrospective cohort study
RCT: Randomized clinical trial
RESAR: Retroperitoneal sarcoma register
RFS: Recurrence-free survival
RPS: Retroperitoneal sarcoma
STS: Soft tissue sarcoma
