# Supplementary material for: Neoadjuvant radiotherapy for resectable retroperitoneal sarcoma: a meta-analysis
Source: Radiat Oncol. 2022 Dec 28;17:215. doi: 10.1186/s13014-022-02159-3 (PMC9795731; doi:10.1186/s13014-022-02159-3)
Supplement: Supplementary file 1 — Additional file 1: Table S1. The quality of enrolled studies. Table S2. Begg’s test for publication bias. Table S3. Univariate/Multivariate Cox proportional HR for outcomes in patients (NRT vs surgery). Fig. S1. Begg’s test for overall survival (OS) of all included studies (a), studies with propensity score matched (PSM) (b), and studies without PSM (c). Begg’s test for recurrence-free survival (RFS) in all included studies (d). [file 13014_2022_2159_MOESM1_ESM.docx]

**Table S1. The quality of enrolled studies**

| **Newcastle Ottawa Score** | | | | | | | | | | |
| --- | --- | --- | --- | --- | --- | --- | --- | --- | --- | --- |
| **Study (author, year)** | **Case selection** | | | | **Comparability** | | **Outcome assessment** | **Follow-up** | **Sufficiency** | **Overall score** |
|  | **1** | **2** | **3** | **4** | **1** | **2** |  |  |  |  |
| Bremjit et al 2014 | √ | √ | √ |  |  | √ | √ | √ | √ | 7 |
| Chouliaras et al 2019 | √ | √ | √ |  | √ | √ | √ | √ | √ | 8 |
| Turner et al 2019 | √ | √ | √ |  | √ | √ | √ | √ | √ | 8 |
| Ecker et al 2016 | √ | √ | √ |  | √ | √ | √ | √ | √ | 8 |
| Kelly et al 2015 | √ | √ | √ |  | √ | √ | √ | √ | √ | 8 |
| Bonvalot et al 2020 | √ | √ | √ |  | √ | √ | √ | √ | √ | 8 |
| Nussbaum et al 2016 | √ | √ | √ |  | √ | √ | √ | √ | √ | 8 |
| Snow et al 2018 | √ | √ | √ |  |  | √ | √ | √ | √ | 7 |
| Lane et al 2015 | √ | √ | √ |  | √ | √ | √ | √ | √ | 8 |
| Bonvalot et al 2009 | √ | √ | √ |  |  | √ | √ | √ | √ | 7 |
| Berger et al 2018 | √ | √ | √ |  |  | √ | √ | √ | √ | 7 |
| Ma et al 2020 | √ | √ | √ |  | √ | √ | √ | √ | √ | 8 |

**Table S2. Begg’s test for publication bias**

| **Categories** | **Bgger’s Test** | |
| --- | --- | --- |
|  | **Z-value** | **Pr>/z/** |
| **OS** | | |
| Neoadjuvant radiotherapy | 0.89 | 0.371 |
| Neoadjuvant radiotherapy_PSM | -0.34 | 1.00 |
| Neoadjuvant radiotherapy_non-PSM | 0.75 | 0.452 |
| **RFS** | | |
| Neoadjuvant radiotherapy | 0.73 | 0.462 |

**Abbreviations:** OS, overall survival; PSM, propensity score matched; RFS, recurrence-free survival

**Table S3. Univariate/Multivariate Cox proportional hazard ratio for outcomes in patients (NRT vs Sur)**

| **Study**  **(Author, Year)** | **Outcome** | | | |
| --- | --- | --- | --- | --- |
|  | **Univariate regression analysis**  **HR (95%CI)** | | **Multivariate regression analysis**  **HR (95%CI)** | |
| Chouliaras et al 2019 | NR | | OS | 1.14 (0.60-2.17) |
|  |  |  | RFS | 0.98 (0.52-1.84) |
|  |  |  | LR | 1.18 (0.51-2.74) |
| Turner et al 2019 | NR | | OS | 0.42 (0.19-0.90) |
|  |  |  | RFS | 0.43 (0.24-0.79) |
| Ecker et al 2016 | NR | | OS | 0.64 (0.42-0.99) |
| Kelly et al 2015 | DSS | 0.46 (0.11-1.95) | DSS | 0.52 (0.12-2.22) |
| Nussbaum et al 2016 | NR | | OS | 0.70 (0.58-0.84) |
| Lane et al 2015 | NR | | OS | 0.30 (0.11-0.82) |
|  |  |  | RFS | 0.34 (0.17-0.69) |
| Bonvalot et al 2020 | NR | | RFS | 1.01 (0.71-1.44) |
| Ma et al 2020 | NR | | OS | 0.88 (0.77-0.99) |
| Bremjit et al 2014 | OS | 0.70 (0.30-1.60) | NR | |
| Snow et al 2018 | OS | 1.00 (0.40-2.70) | NR | |
|  | RFS | 0.33 (0.13-0.84) |  |  |
| Bonvalot et al 2009 | LR | 0.64 (0.45-0.90) | NR | |
| Berger et al 2018 | NR | | OS | 0.89 (0.69-1.14) |

**Abbreviations:** CI, confidence interval; DSS, disease-specific survival; HR, hazard ratio; LR, local recurrence; NR, not reported; OS, overall survival; RFS, recurrence-free survival; NRT, neoadjuvant radiotherapy, Sur, surgery

**Figure S1 Begg’s test for overall survival (OS) of all included studies (a), studies with propensity score matched (PSM) (b), and studies without PSM (c). Begg’s test for recurrence-free survival (RFS) in all included studies (d).

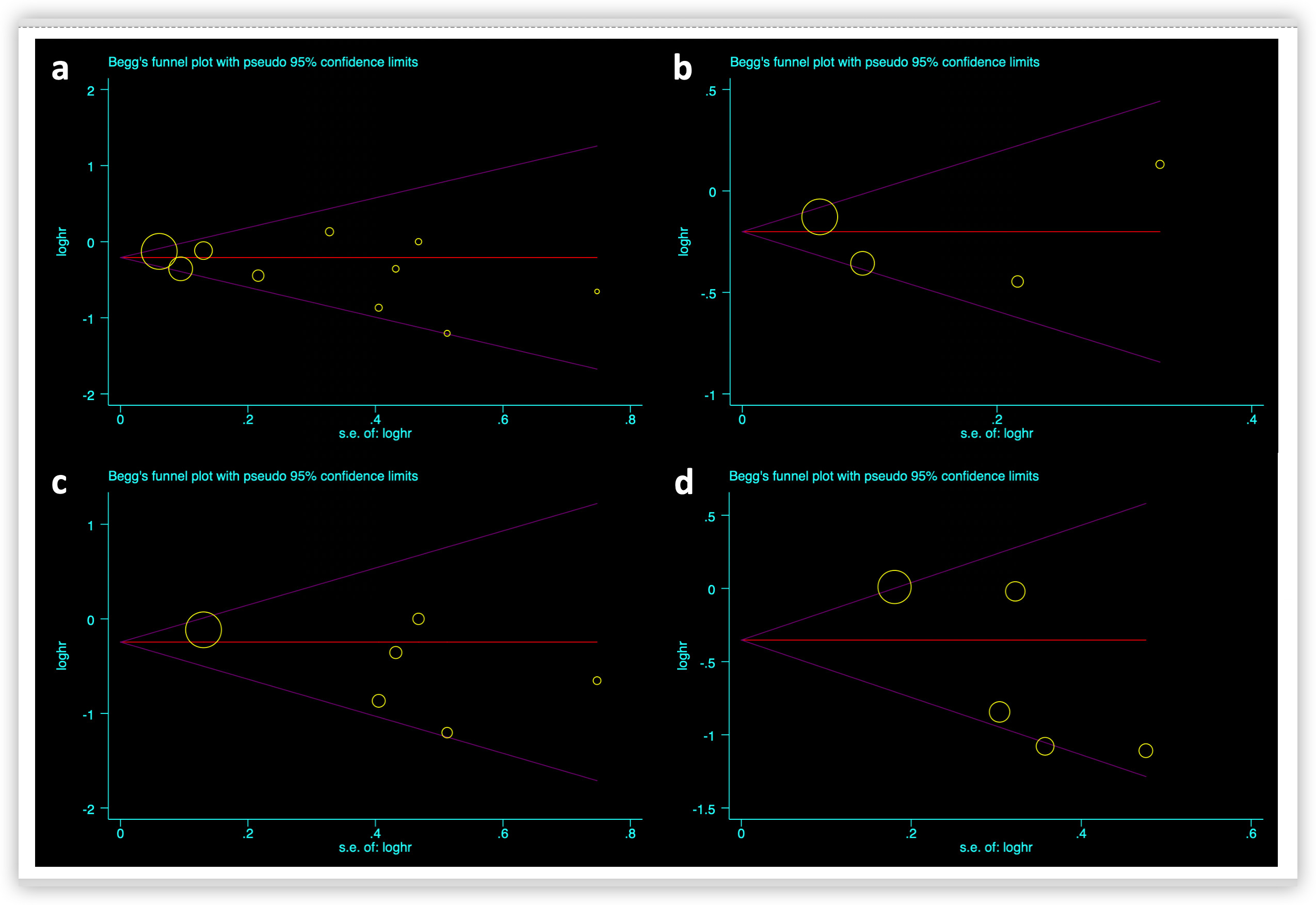
**
